# Distinct Presentations of Langerhans Cell Histiocytosis in Children: A Case Series

**DOI:** 10.1155/crom/5583430

**Published:** 2025-10-30

**Authors:** Gashaw Arega, Kirubel Asmelash, Michael A. Negussie, Abebe Mekonnen

**Affiliations:** ^1^Department of Pediatrics and Child Health, College of Health Sciences, Addis Ababa University, Addis Ababa, Ethiopia; ^2^School of Medicine, College of Health Sciences, Addis Ababa University, Addis Ababa, Ethiopia; ^3^Department of Radiology, College of Health Sciences, Addis Ababa University, Addis Ababa, Ethiopia

**Keywords:** case series, CD1a positivity, Langerhans cell histiocytosis, pediatric

## Abstract

Langerhans cell histiocytosis (LCH) is a rare pediatric histiocytic disorder characterized by diverse clinical manifestations, ranging from isolated lesions to severe multisystem involvement. This case series presents three distinct presentations observed in children. The first case involved a 4-year-old female presenting with generalized lymphadenopathy, polyuria, polydipsia, bilateral vision loss, and systemic symptoms, indicative of significant pituitary and multisystem involvement. Imaging revealed lesions involving the pituitary gland, hypothalamus, and sphenoid sinus. The second case described a 10-year-old male experiencing respiratory distress, significant weight loss, polyuria, and multiple lytic bone lesions. Diagnostic imaging identified extensive colonic involvement, bilateral hydronephrosis, and pulmonary lesions, emphasizing unusual systemic features. The third patient, an 18-month-old child, initially presented with persistent respiratory symptoms, a diffuse rash, severe acute malnutrition, and hepatomegaly and was initially misdiagnosed as having tuberculosis. Later imaging studies revealed extensive pulmonary cystic lesions. Immunohistochemistry from the tissue biopsy demonstrated CD1a positivity, confirming LCH diagnoses. Treatment strategies included standard induction protocols with vinblastine and corticosteroids. These cases show how LCH can present in many different ways in pediatric patients, often in unexpected patterns. Early recognition, thorough imaging, and histological confirmation are crucial for accurate diagnosis. Being aware of the wide range of symptoms can help ensure prompt treatment and better outcomes for this serious but manageable condition.

## 1. Introduction

Histiocytic disorders are diverse conditions classified based on cellular origin, distribution, and molecular lesions [1]. Langerhans cell histiocytosis (LCH), the most common pediatric histiocytic disorder, presents variably, ranging from isolated lesions to life-threatening multisystem disease [1, 2]. Typically, LCH lesions contain CD1a+/CD207+ dendritic cells, lymphocytes, eosinophils, and macrophages [1].

LCH commonly affects the skeleton, skin, and pituitary, with symptoms such as skin rash, bone pain, soft tissue swelling, fever, and weight loss [2, 3]. The BRAFV600E mutation, found in over half of cases, particularly in severe forms, predicts a higher risk of treatment failure [2, 4]. Incidence peaks in early childhood, with variation among ethnic groups and links to environmental and infectious factors [2, 3, 5].

Diagnosis typically involves CD1a/CD207 positivity, supported by imaging and molecular detection of BRAFV600E mutations [2, 3]. Prognosis and treatment outcomes vary with disease severity, ranging from excellent survival in low-risk cases to moderate risk in high-risk cases, with notable long-term complications [3, 6].

Herein, we present three pediatric cases of LCH, each demonstrating distinct clinical presentations, diagnostic findings, and therapeutic approaches.

## 2. Case Presentation

### 2.1. Case 1

A 4-year-old female presented with a 2-month history of gradually enlarging swellings over the neck, axilla, and inguinal regions. Associated symptoms included intermittent low-grade fever, decreased appetite, and significant but unquantified weight loss. One month prior to presentation, she developed polyuria, polydipsia, and bilateral vision loss. On examination, the child appeared comfortable with normal anthropometric measures. Multiple nontender lymphadenopathies were observed in the cervical, submental, submandibular, axillary, and inguinal regions, with the largest node measuring 3 × 3 cm in the submandibular area. Bilateral vision loss was confirmed.

Laboratory investigations, including complete blood count, renal and liver function tests, lactate dehydrogenase, uric acid, and electrolytes, were unremarkable except for an elevated ESR (45 mm/h). Thyroid function was within normal limits (TSH: 2.53 mIU/L, FT4: 0.85 ng/dL), and serum cortisol was 128 ng/mL. Bone marrow aspiration and peripheral blood morphology demonstrated trilineage hematopoiesis. Chest x-ray showed hilar lymphadenopathy (LAP), and abdominal ultrasound revealed multiple splenic nodules (largest 2.3 × 1.7 cm) and distal ileal bowel thickening.

**Figure 1:**
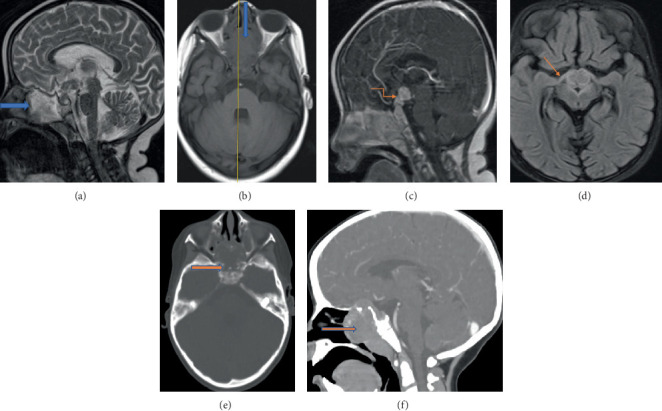
(a) There is a heterogeneously hyperintense lesion in the sphenoid sinus on sagittal T2W, (b) with intermediate signal intensity on T1W, associated with a nodular intermediate signal intensity lesion involving the pituitary, infundibulum, and hypothalamus. (c) Both the sphenoid sinus and intracranial sellar and suprasellar components show homogeneous contrast enhancement on sagittal T1+C. (d) There is optic tract and optic chiasm edema on axial FLAIR. (e) Axial CT scan in the bone window shows midline basal skull destruction and otomastoid air cell opacification, (f) while the sphenoid sinus and intracranial soft tissue components demonstrate enhancement on the soft tissue window of sagittal CT MPR (f).

Initial CT scans of the chest and abdomen demonstrated multiple hypoenhancing splenic lesions with cervical, mediastinal, and para-aortic LAP. Brain CT and MRI revealed sphenoid sinus and pituitary lesions with sellar and suprasellar soft tissue components, associated with midline basal skull destruction and optic tract involvement (Figure 1). A lymph node biopsy showed sheets of atypical histiocytic cells with irregularly folded and grooved nuclei, admixed with eosinophils and lymphocytes, confirming the diagnosis of LCH. Follow-up CT scans 6 weeks after initiation of vinblastine and prednisolone therapy demonstrated significant radiological improvement.

### 2.2. Case 2

A 10-year-old male presented with a 3-month history of easy fatigability and significant but unquantified weight loss. Two months prior, he developed a dry intermittent cough, low-grade intermittent fever, and increased frequency of urination without dysuria or hematuria. On examination, the child was in respiratory distress with severe malnutrition (BMI-for-age < −3 *Z* score), pale conjunctiva, and multiple firm, nonmatted, nontender lymphadenopathies in the cervical, axillary, and submandibular regions. Integumentary examination revealed whitish, flaky plaques on the soles of both feet.

**Figure 2:**
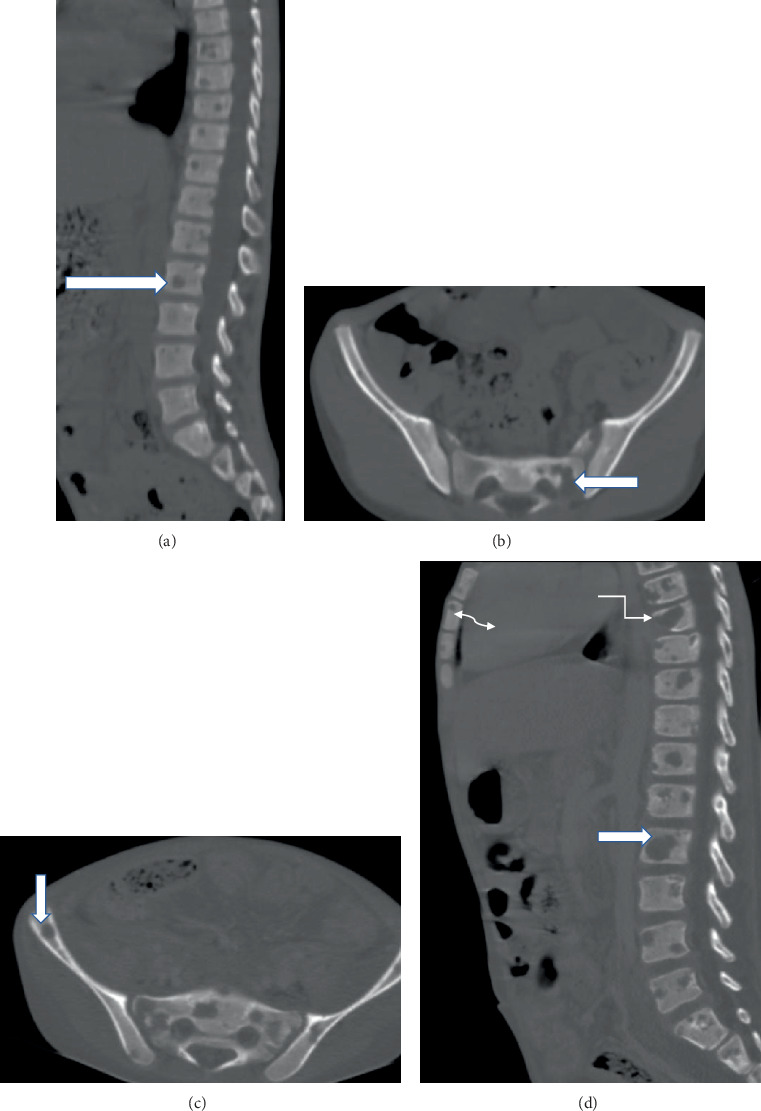
(a, b) There are multiple lytic lesions involving the lower thoracic, all lumbar, and sacral vertebrae, as well as the iliac bones, on thoracolumbar and pelvic CT (bone window) (indicated by horizontal and vertical fat arrows) taken on the first visit. (c, d) Taken after 2 months, they show an increase in the number and size of the lytic lesions and the development of a pathologic wedge compression fracture of the Th8 vertebra (elbow arrow) and a lesion on the included sternum.

Laboratory tests showed leukocytosis (WBC: 18,000 cells/*μ*L, neutrophils: 85%) and microcytic anemia (HGB: 8.4 g/dL, MCV: 70 fL). Liver and renal function tests and electrolytes were within normal limits. Bone marrow aspiration demonstrated trilineage hematopoiesis. Imaging revealed an unremarkable skull x-ray and abdominal CT showing long-segment colonic wall thickening with mesenteric fat stranding and bilateral Grade 3 hydronephrosis. Thoracolumbar and pelvic CT demonstrated multiple lytic bone lesions and intra-abdominal lymphadenopathies (Figure 2).

The patient received nutritional rehabilitation, antibiotic therapy, and desmopressin for persistent polyuria. He was subsequently initiated on an LCH induction protocol with vinblastine and prednisolone, showing significant clinical improvement.

### 2.3. Case 3

A 1-year-and-6-month-old child presented with a 5-month history of dry intermittent cough, fast breathing, and grunting respirations. A diffuse maculopapular, hypopigmented rash was noted over the scalp, face, and trunk. Initially treated empirically for tuberculosis without improvement, the child later presented in severe respiratory distress (SpO_2_: 72%) with severe malnutrition (weight-for-height < −3 *Z* score) and hepatomegaly (6 cm below the right costal margin).

Laboratory findings revealed transient elevation of liver enzymes (approximately 2× normal), slight hypercalcemia (11 mg/dL), and elevated phosphorus (6.3 mg/dL). Lymph node biopsy demonstrated sheets of atypical histiocytic cells with irregularly folded and grooved nuclei, admixed with eosinophils and lymphocytes. Immunohistochemistry confirmed positivity for CD1a. Echocardiography and abdominal ultrasound were unremarkable at presentation.

**Figure 3:**
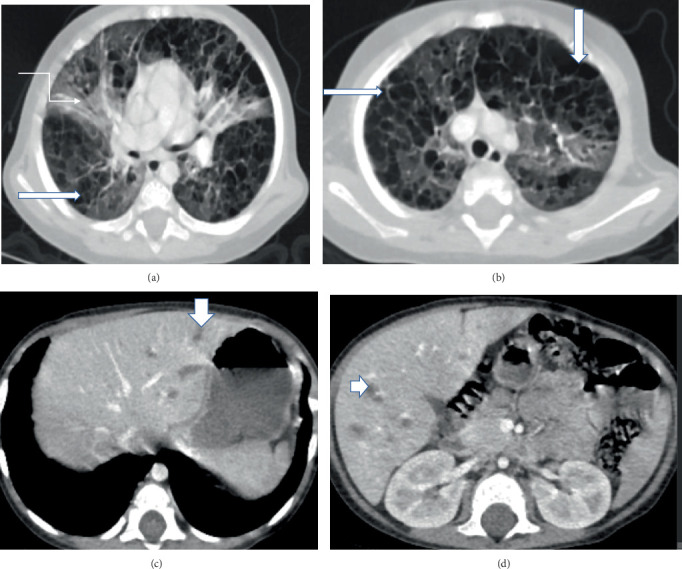
(a, b) Chest CT scan (lung window) shows diffuse, multiple, confluent, and bizarre-shaped air-containing cysts with perceptible walls and septal thickening (horizontal and vertical fat arrows) in all lobes, along with a few superimposed segmental ground-glass attenuations (elbow connector arrow). (c, d) There are scattered multiple hypodense lesions involving both liver lobes on upper abdomen CT (shown by arrow down and arrow right).

Chest CT revealed diffuse, multiple, confluent, and bizarre-shaped air-containing cysts with perceptible walls and septal thickening in all lobes, along with a few superimposed segmental ground-glass attenuations. Upper abdominal CT showed scattered hypodense lesions involving both liver lobes (Figure 3).

The patient received oxygen therapy, nutritional rehabilitation, and was initiated on an LCH induction regimen with prednisolone and vinblastine.

## 3. Discussion

Although LCH is more common in children than adults, its incidence remains low, approximately six cases per million, categorizing it as a rare condition. Therefore, it was particularly noteworthy to observe a cluster of cases at our hospital within a single month. LCH can involve any organ, with the skeleton (80%), skin (33%), and pituitary gland (25%) being the most frequently affected [3].

The first patient presented with bilateral vision loss, multiple systemic symptoms, and generalized LAP, strongly suggesting pituitary involvement. Brain MRI revealed nodular, homogeneously enhancing lesions involving the pituitary gland, pituitary stalk, hypothalamus, and an expansile enhancing soft tissue lesion within the sphenoid sinus, accompanied by adjacent bone destruction.

Central diabetes insipidus (DI) occurs in approximately 25% of LCH cases, predominantly affecting children and frequently associated with orbit and skull involvement. Most DI cases initially present alongside systemic disease but can occasionally manifest as isolated pituitary involvement. While posterior pituitary involvement is common, additional endocrine manifestations of LCH include growth hormone deficiency, adrenal insufficiency, hyperprolactinemia, and hypogonadism due to hypothalamic infiltration affecting the anterior pituitary gland [3].

LCH is among the differential diagnoses for pituitary stalk lesions, which may present with hormonal deficiencies, precocious puberty, or nonspecific symptoms such as headache and visual disturbances [7]. Bone destruction observed on imaging aligns with typical findings in LCH, often demonstrated as lytic bone lesions [2]. However, significant lymph node involvement, as seen in the first case, is less common.

The second and third cases presented primarily with respiratory symptoms, including dry cough and respiratory distress, accompanied by nonspecific systemic manifestations. The third patient also displayed significant dermatological involvement, with a diffuse rash predominantly affecting the scalp and face. Skin rash, fever, and weight loss are among the most common initial presentations of LCH [2, 3].

Though prominent pulmonary involvement is relatively uncommon, both the second and third cases exhibited significant respiratory findings. Chest CT in the second patient demonstrated multiple rib lytic lesions and left lung ground-glass opacity, while the third patient's imaging revealed bilateral lung parenchymal cystic changes, both indicative of pulmonary LCH. Pulmonary involvement in LCH can be severe, leading to a chronic debilitating condition and potential spontaneous pneumothorax [1]. Fortunately, both patients improved and were discharged without supplemental oxygen.

Definitive diagnosis of LCH requires positive CD1a and/or CD207 (Langerin) immunostaining of affected cells [3]. In rare instances, when biopsies pose significant risks, clinicians must carefully weigh the risk–benefit ratio before proceeding [3].

Standard first-line treatment includes vincristine and prednisolone combination therapy. The initial induction therapy comprises one or two 6-week courses: vindesine administered as a 3 mg/m^2^ IV bolus once weekly for 6 weeks, combined with prednisone at 40 mg/m^2^/day orally for 4 weeks, followed by a weekly dose reduction over 2 weeks. Depending on treatment response assessed at 6 weeks, one or two induction cycles are given. Maintenance therapy involves vindesine (3 mg/m^2^ IV bolus every 3 weeks), prednisone (40 mg/m^2^ orally on Days 1–5 every 3 weeks), and 6-mercaptopurine (50 mg/m^2^ orally daily) for single-system patients with CNS-risk lesions and multisystem patients. The total duration of first-line therapy is typically 12 months [8].

All three patients were initiated on vinblastine and steroid induction therapy. Following the 6-week response assessment, the first and second patients proceeded to the continuation phase, while the third patient required initiation of salvage therapy.

## 4. Conclusion

LCH in children can present with a wide range of symptoms, from isolated lesions to severe multisystem disease. Recognizing and diagnosing atypical or multisystem cases early is important for guiding treatment. A thorough evaluation is needed in children with LAP, respiratory symptoms, or unexplained systemic findings, even when typical imaging patterns are not present.

## Data Availability

The data that support the findings of this study are available from the corresponding author upon reasonable request.
